# Parliament and Publications

**Published:** 1910-10-01

**Authors:** 


### Parliament and Publications.

### SIGHT TESTS

A report on the Sight Tests used in the Mercantile Marine, which has been issued as a Parliamentary Paper, states that during 1909 the total number of candidates examined was 6,084. Of these, 6,028 candidates passed in form vision, and 56 failed. 5,942 candidates were successful in the colour vision tests, and 86 failed; 15 of the latter passed on appeal. No case of failure to pass the colour-ignorance test has been reported. The Board of Trade have decided that after January 1, 1914, the standard of form vision shall be raised, so that candidates will thereafter be required to possess full normal vision in one eye and at least half-normal vision in the other.—" Sight Tests." Cd. 5256. Price 3d.

### PUBLIC HEALTH IN IRELAND.

The annual report of the Local Government Board for Ireland for the year ended March 31, 1910, contains much information of medical interest. With regard to the notification of tuberculosis, provided for by the Tuberculosis Prevention Act, 1908, which came into force on July 1, 1909, the Board is of opinion that the progress so far made in regard to the adoption of notification is not unsatisfactory, but the hope is expressed that, before long, steps will be taken to introduce the compulsory notification of tuberculosis in all the larger urban districts. Such a course is specially desirable in the county boroughs of Cork and Waterford, where the incidence of phthisis is particularly heavy. The number of cures notified in Belfast County Borough from November 2, 1909, to April 2, 1910, was 497 (309 females and 188 males); and in Dublin County Borough from October 1, 1909, to March 31, 1910, 588 (245 females and 343 males). It may be pointed out that it is left to the discretion of each sanitary authority to avail themselves of the principle of compulsory notification, and that the Act limits notification to cases of tuberculosis which are a danger to others, owing to the infectious nature of the discharge from the patients.

The country has been entirely free from smallpox during the year. The notifications of typhus fever numbered 182, as compared with 162 in the previous year. Enteric fever was less prevalent than in the preceding year. Scarlatina appeared to an exceptional extent, the spread of the disease being facilitated by its mild character, which led to the primary cases escaping recognition.

As to vaccination, the number of successful vaccinations performed by the medical officers of workhouses and dispensary districts, and at the Vaccine Institute, was 82,046. An agitation was started at Enniscorthy against vaccination, and had the effect of bringing it almost to a standstill within the union. The number of tubes of glycerinated calf lymph issued was 112,123, and the activity and purity of the lymph is referred to in the highest terms. It has been decided not to vaccinate calves at the National Vaccine Institute during the hot weather, but to accumulate in cold storage all the vaccine collected during the cold months of the year.

The report of the pharmacist to the Local Government Board shows that the number of samples of drugs reported upon was 8,900, of which 112 were below standard. The total expenditure of medicines and appliances exceeded the amount of the previous year by the sum of £665. Of this increase, however, £546 was due to the extra duty on spirit, and the consequent increased prices charged for drugs into which spirit entered as an ingredient. " Annual Report of the Local Government Board for Ireland" (Cd. 5319). Price 1s. 9d.

